# SDF1-A Facilitates Lin−/Sca1+ Cell Homing following Murine Experimental Cerebral Ischemia

**DOI:** 10.1371/journal.pone.0085615

**Published:** 2014-01-20

**Authors:** J. Mocco, Aqeela Afzal, Saeed Ansari, Annemarie Wolfe, Kenneth Caldwell, E S. Connolly, Edward W. Scott

**Affiliations:** 1 Department of Neurological Surgery, Vanderbilt University, Nashville, Tennessee, United States of America; 2 Department of Neurosurgery, University of Florida, Gainesville, Florida, United States of America; 3 Department of Neurological Surgery, Columbia University, New York, New York, United States of America; 4 Department of Molecular Genetics and Microbiology, University of Florida, Gainesville, Florida, United States of America; University of Colorado, Denver, United States of America

## Abstract

**Background:**

Hematopoietic stem cells mobilize to the peripheral circulation in response to stroke. However, the mechanism by which the brain initiates this mobilization is uncharacterized.

**Methods:**

Animals underwent a murine intraluminal filament model of focal cerebral ischemia and the SDF1-A pathway was evaluated in a blinded manner via serum and brain SDF1-A level assessment, Lin−/Sca1+ cell mobilization quantification, and exogenous cell migration confirmation; all with or without SDF1-A blockade.

**Results:**

Bone marrow demonstrated a significant increase in Lin−/Sca1+ cell counts at 24 hrs (272±60%; P<0.05 vs sham). Mobilization of Lin−/Sca1+ cells to blood was significantly elevated at 24 hrs (607±159%; P<0.05). Serum SDF1-A levels were significant at 24 hrs (Sham (103±14), 4 hrs (94±20%, p = NS) and 24 hrs (130±17; p<0.05)). Brain SDF1-A levels were significantly elevated at both 4 hrs and 24 hrs (113±7 pg/ml and 112±10 pg/ml, respectively; p<0.05 versus sham 76±11 pg/ml). Following administration of an SDF1-A antibody, Lin−/Sca1+ cells failed to mobilize to peripheral blood following stroke, despite continued up regulation in bone marrow (stroke bone marrow cell count: 536±65, blood cell count: 127±24; p<0.05 versus placebo). Exogenously administered Lin−/Sca1+ cells resulted in a significant reduction in infarct volume: 42±5% (stroke alone), versus 21±15% (Stroke+Lin−/Sca1+ cells), and administration of an SDF1-A antibody concomitant to exogenous administration of the Lin−/Sca1+ cells prevented this reduction. Following stroke, exogenously administered Lin−/Sca1+ FISH positive cells were significantly reduced when administered concomitant to an SDF1-A antibody as compared to without SDF1-A antibody (10±4 vs 0.7±1, p<0.05).

**Conclusions:**

SDF1-A appears to play a critical role in modulating Lin−/Sca1+ cell migration to ischemic brain.

## Introduction

Each year approximately 795,000 Americans experience a new or recurrent stroke. [1] Increasing levels of circulating Hematopoietic Stem Cells (HSC)/Hematopoietic Progenitor Cells (HPC) have recently been demonstrated to correlate with improved neurological function following stroke, suggesting a potentially critical role for HSC/HPC’s in limiting stroke injury and/or facilitating post-stroke recovery. [2]–[6] HSC/HPC’s are circulating bone marrow derived mononuclear cells that reside in the adult bone marrow and have the unique ability to self renew and differentiate into multiple lineages. [7] HSC/HPC’s are known to mobilize to the peripheral circulation from bone marrow in response to stroke. [8]–[10] Additionally, it has been suggested that stroke recovery can be augmented with angiogenic blood vessel formation. [11] Mobilized HSC/HPC are recruited to the site of injury and can subsequently contribute to angiogenesis. [11] Chronic heart disease [12] and hind limb ischemic [13] studies have shown promising therapeutic results from mobilized HSC/HPC.

Stromal Derived Growth Factor-1 Alpha (SDF1-A) is localized to chromosome 10q11.1 [14] and is highly conserved between species. [15] SDF1-A belongs to the CXC family of chemokines and was originally described as a pre B cell growth stimulating factor. [15] SDF1-A is a ligand for CXCR4, a G protein coupled receptor, and their interaction mediates a chemotactic response followed by cell migration. [16] CXCR4 is expressed on several cell types and was the only known receptor for SDF1-A to induce vasculogenesis, [17] hematopoiesis, [18] chemotaxis, [19] and metastasis [19] until another receptor, CXCR7 was recently discovered. [20] SDF1-A and CXCR4 have been shown to regulate trafficking of HSC/HPC in response to non-cerebral injury. [21]–[23] Additionally, hematopoietic stem cells have also been shown to mobilize from the bone marrow to the blood in response to injury. [19] De Falco et al. demonstrated that ischemic blood vessels in a hind limb ischemia model release SDF1-A, which, in turn, triggers the mobilization of the HSC from the bone marrow (a distant healthy niche) to the peripheral blood. [24]–[27] Once in the circulation, the HSC can differentiate into myeloid cells, lymphocytes, erythrocytes, platelets or endothelial progenitor cells. [28] In the myocardium, [29] HSC/HPC’s have been shown to home towards SDF1-A released from ischemic regions [27] where they mature into endothelial cells and contribute to resident vasculature repair. [19].

SDF1-A is a powerful chemo attractant [30] and is expressed by several tissues in the body including bone marrow, [31] liver, [32] kidney [33] and the central nervous system. [34] SDF1-A is expressed in tissues during development [35] and in adulthood. [25] SDF1-A has been implicated in the homing of exogenously administered (IV or direct intraparenchymal injection) bone marrow derived mesenchymal stem cells (BSMC’s) to ischemic brain in rats. [36], [37] However, the application of these data to humans was brought into question, when, in a murine model – the most common species evaluated for many stroke therapeutics, exogenously administered human BSMC’s failed to ‘home’ to the ischemic brain. [38] Furthermore, these studies did not evaluate endogenous HSC/HPC mobilization and the influence of SDF1-A axis on this mobilization or subsequent potential homing. We hypothesized that, following murine experimental cerebral ischemia, SDF1-A may direct an increased mobilization of HSC/HPC from the bone marrow to the peripheral blood. The HSC/HPC may subsequently home to the area of cerebral ischemia, possibly facilitating reparative mechanisms.

## Methods

### Animals

Nine-week-old C57/BL/6 male (unless otherwise specified) mice were obtained from Harlan Laboratories (Tamps, Florida). This study was carried out in strict accordance with the recommendation in the guide for the care and use of Laboratory Animals of the National Institute of health. The protocol was approved by the Committee on the Ethics of Animal Experiments of Vanderbilt University (Permit Number: A3227-01). Animals were kept under specific conditions according to protocols approved by the Institutional Animal Care and Usage Committee and all efforts were made to minimize suffering.

### Intraluminal Filament Model of Stroke

A heat blunted nylon monofilament 7-0 suture, 11–13 mm in length was introduced through the external carotid artery and then into the common carotid where the filament was fed into the internal carotid artery until it blocked the middle cerebral artery. Sham animals were treated the same as the experimental animals, minus introduction of the monofilament suture. Occlusion was confirmed by laser Doppler flowmetry (Perimed, Ardmore, PA) and was maintained for 45 minutes. Middle cerebral artery occlusion was considered to be technically adequate at ≥80% reduction in cerebral blood flow was observed immediately following placement of the occluding catheter. Animal temperature was carefully controlled using a water heated circulating pad using a rectal probe (World Precision Instruments, Sarasota, Fl).

Following surgery, animals were scored at time of sacrifice to obtain neurologic deficit score and confirm cerebral ischemia, as previously described. [39] An animal with no observable neurological deficit was given a score of 0; if the animal failed to extend the contralateral paw, it was given a score of 1; an animal circling to the right was given a score of 2; if the animal fell over on the contralateral side when attempting to walk, it was given a score of 3; if an animal did not exhibit any spontaneous motor activity, it was given a score of 4.

When evaluated, cerebral infarct volume was calculated using digital planimetric analysis of 2 mm sectioned 2,3,5-Triphenyltetrazolium chloride (TTC) stained brains, as previously described. [39] Briefly, Brain tissue was sectioned coronally at 2 mm intervals and the sections placed in TTC for 30 minutes at 37°C. Digital images were obtained for each section and for each section the area of infarct, area of entire ipsilateral hemisphere and area of contralateral hemisphere were calculated using Image J. The respective volumes were then calculated by summing the area of the values multiplied by the thickness. To obtain the final infarct volume corrected for edema, the following formula was used: [contralateral hemisphere volume –(ipsilateral hemisphere volume-infarct volume)]/contralateral hemisphere×100.

### Do Lin−/Sca1+ Cell Levels Respond to Stroke?

Eighteen mice were used as sham controls (4 hours, n = 9; 24 hours, n = 9) and 18 mice underwent surgery (n = 9 for 4 hours; n = 9 for 24 hours) to evaluate serum and bone marrow HSC/HPC (Lin−/Sca1+ cells) response to stroke. At the time of sacrifice, blood from each mouse was harvested along with the hind leg bones (femurs). The mononuclear cells from the blood and the bone marrow from the above mentioned cohorts were washed with a phosphate buffered saline containing 2% fetal bovine serum (Fisher Scientific, Pittsburg, PA). The washed mononuclear cell layer from each blood and bone marrow sample was then re-suspended in buffer and Lin−/Sca1+ selection kits (Stem Cell Technologies; Vancouver, CA) used to obtain the Lin−/Sca1+ cells from the samples. The enriched cells were counted using a hemacytometer (Fisher Scientific; Pittsburg, PA). Each sample was counted three times. The number of cells counted was adjusted for volume and reported as total number of cells obtained for each sample.

### Do SDF1-A Levels Increase in Serum and Brain?

Sixteen animals were divided into 3 cohorts (sham (n = 6), 4 hours (n = 5) and 24 hours (n = 5) hours post stroke surgery) for SDF1-A analysis in serum and brain tissue. Two hundred microliters of blood was obtained from mice at the 4 and 24-hour time points. The blood was allowed to clot at room temperature, spun at 2000 RPM and the serum removed for analysis by SDF1-A ELISA per manufacturer’s protocol (R&D Systems, Minnepaolis, MN). Brain tissue was homogenized in RIPA buffer with protease inhibitors (Pierce Biotechnology, Rockford, IL) and quantified for protein using a BCA protein assay (Pierce Biotechnology, Rockford, IL). The homogenized samples (normalized for protein) were analyzed for SDF1-A levels using an ELISA per manufacturer’s protocol (R&D Systems, Minnepaolis, MN).

### Does SDF1-A Blockade Prevent Lin−/Sca1+ Cell Mobilization?

To evaluate the relative contribution of SDF1-A to the Lin−/Sca1+ cell response, commercially available SDF1-A neutralizing antibody (100 ug dose; R&D Systems, Minneapolis, MN), or a PBS (vehicle) control was administered via IP injection one day prior to surgery and again immediately following surgery. Eighteen animals were divided into 2 groups (n = 9 each): stroke+placebo and stroke+SDF1-A antibody. Lin−/Sca1+ cells were enriched and counted from the bone marrow and blood as described above.

### Does SDF1-A Blockade Abrogate Homing?

Sixteen mice were divided into stroke+placebo (n = 9) and stroke+ Lin−/Sca1+ cells (n = 7) cohorts. Another cohort of 14 animals was divided into stroke+SDF1-A antibody+placebo (n = 9), stroke+SDF1-A antibody+ Lin−/Sca1+ cells (n = 5). For those animals receiving Lin−/Sca1+ cells injection, the cells were enriched from a separate group of control mice (n = 24) and injected IV at reperfusion. Infarct volumes were calculated at 24 hours post stroke.

Furthermore, Lin−/Sca1+ cells were enriched from male donor mice (n = 16) and injected IV at reperfusion into 2 cohorts of female mice (stroke+placebo+ Lin−/Sca1+ cells n = 4; stroke+SDF1-A antibody+ Lin−/Sca1+ cells n = 4). The animals were sacrificed at 24 hours post stroke and their brain removed for FISH analysis. For FISH analysis, Paraformaldehyde fixed frozen mouse brains were cut at 50 um through the area of infarct. Slides were air dried overnight, washed 2×5 minutes in PBS and then rinsed briefly in ddH2O. Antigen retrieval was performed by immersing slides in 1 M sodium thiocyanate for 30 minutes at 95°C. The slides were removed from the retrieval solution and rinsed thoroughly in ddH2O before digestion in 4 mg/ml porcine pepsin (Sigma Aldrich, St Louis, MO) in 1XPBS at pH 2.0 for 10 minutes. Slides were rinsed 1 min in water, 1 min in 2X Saline Sodium Citrate Buffer (SSC) and then dehydrated in increasing concentrations of Ethanol before probe was applied. Mouse chromosome X (orange) and Y (green) were applied to the cells following the manufacturer’s directions (Cambio Ltd, Cambridge, UK). Slides were denatured and hybridized using a Hybrite oven (Vysis, Downer’s Grove, IL) overnight at 37°C. Slides were washed at 46°C in 50% formamide/2XSSC 3X 7 minutes, in 2X SSC for 5 minutes and in 4X SSC+0.1% Igepal for 5 minutes. Slides were permitted to air dry in the dark for 10 minutes before cover slipping with DAPI Vectorshield (Vector labs, Burlingame, CA). Slides were documented with an Olympus BX10 Microscope (Hunt Optics & Imaging, INC., Pittsburg, PA). The number of male (Y) chromosome positive cells counted and the sum of cells counted per high power field are shown.

### Analysis

The technician performing the surgeries, and all subsequent analysis, was completed with total blinding to experimental cohort across all experiments. All statistical analysis was performed using the Students t-test, Mann-Whitney Test or ANOVA with a post hoc Newman-Keuls Multiple Comparison test (GraphPad Software version 5, Inc. La Jolla, CA, USA). Mean values are reported as mean±SD, and a p value of less than 0.05 was considered to be significant and is indicated on subsequent graphs with an asterisk.

## Results

Cortical blood flow (CBF) measured using a Trans-cranial doppler after middle cerebral artery occlusion decreased by at least 80% in all animals. Animals that underwent stroke surgery had a consistently higher neurological deficit score compared to sham animals (all sham animals had a neurologic deficit score of 0). For early (4 hour) stroke cohort analysis neurologic deficit was used to confirm stroke, as TTC staining is inconsistent at such early assessments. Across all experiments no significant difference was observed in the 4 hour versus 24-hour cohorts’ neurological deficit scores.

### Do Lin−/Sca1+ Cell Levels Respond to Stroke?

**Figure 1:**
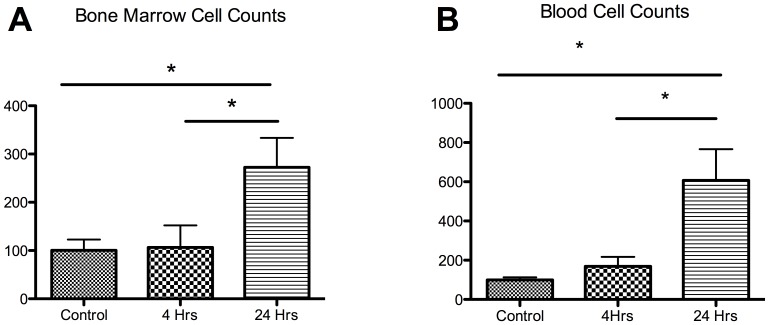
Enumeration of bone marrow and blood Lin−/Sca1+ cell numbers following stroke. (A) Twenty-four hour post stroke mice show a marked increase in the number of Lin−/Sca1+ cells present in the bone marrow compared to controls. (B) Twenty-four hour post stroke mice also showed a marked increase in the mobilization of these cells to the blood. * P<0.05.

Analysis of the ability of Lin−/Sca1+ cells to mobilize from the bone marrow to the peripheral blood following stroke (Figure 1) demonstrate that, following murine stroke, bone marrow production of Lin−/Sca1+ cells was minimal at 4 hours (106±46% versus sham), but achieved a highly significant increase by 24 hours (272±60% versus sham). Mobilization of the Lin−/Sca1+ cells to the peripheral blood was also non-significantly elevated at 4 hours (169±48% versus sham) and significantly elevated at 24 hours (607±159% versus sham; P<0.05).

### Do SDF1-A Levels Increase in Serum and Brain?

**Figure 2:**
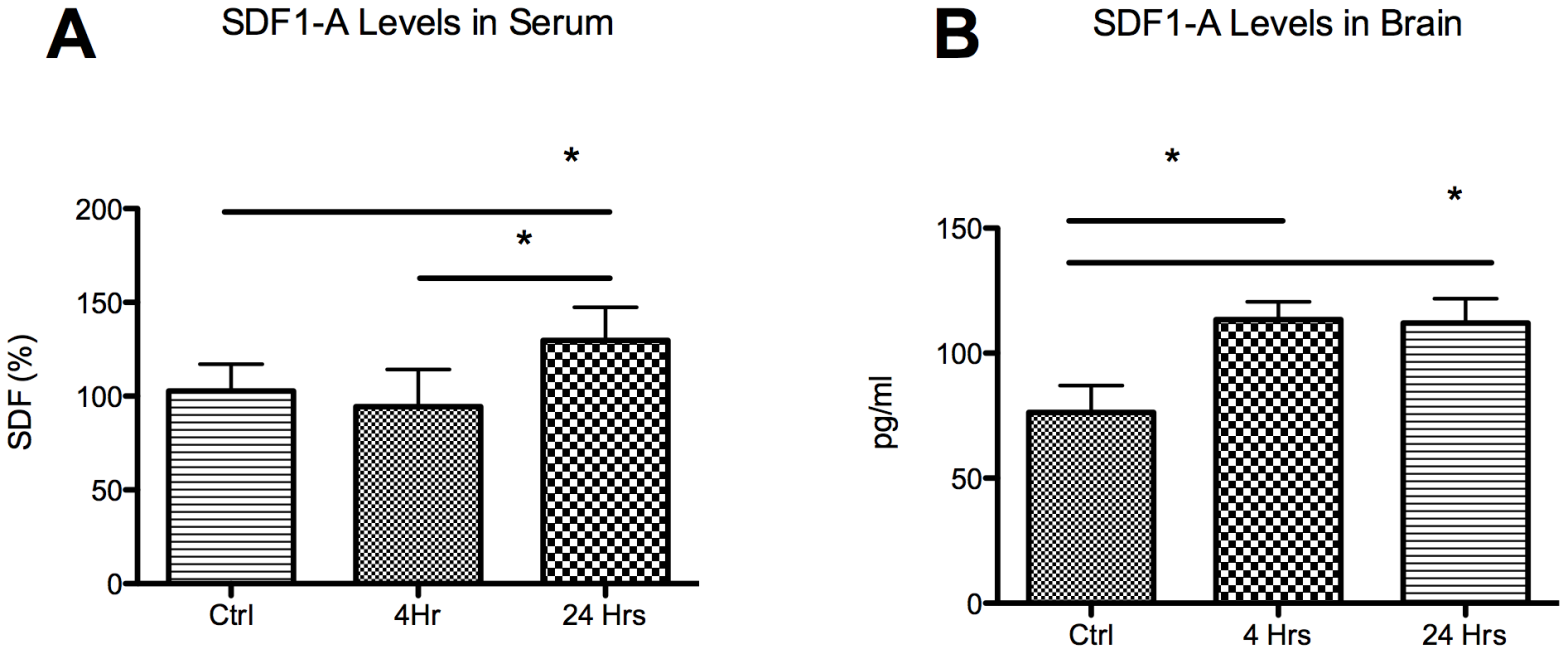
SDF1-A levels in serum and brain. (A) Serum SDF1-A levels reached significance at 24 hours post surgery. (B) Brain tissue levels of SDF1-A demonstrated significant elevations at both 4 and 24 hours post stroke compared to sham animals. *P<0.05.

ELISA (Figure 2A) demonstrated that the SDF1-A levels in the mouse serum were not significantly elevated at 4 hours, but were significantly elevated by 24 hours post ischemia (102±14%, 94±20% and 130±18%, Control, 4 hours and 24 hours respectively; 4 hrs vs control: P = NS, 24 hrs vs control: p<0.05). Brain SDF1-A level analysis (Figure 2B) demonstrated significant elevation at both 4 and 24 hours post stroke (113±7 pg/ml and 112±10 pg/ml, respectively; p<0.05 for both versus control 76±11 pg/ml).

### Does SDF1-A Blockade Prevent Normal Lin−/Sca1+ Cell Mobilization?

**Figure 3:**
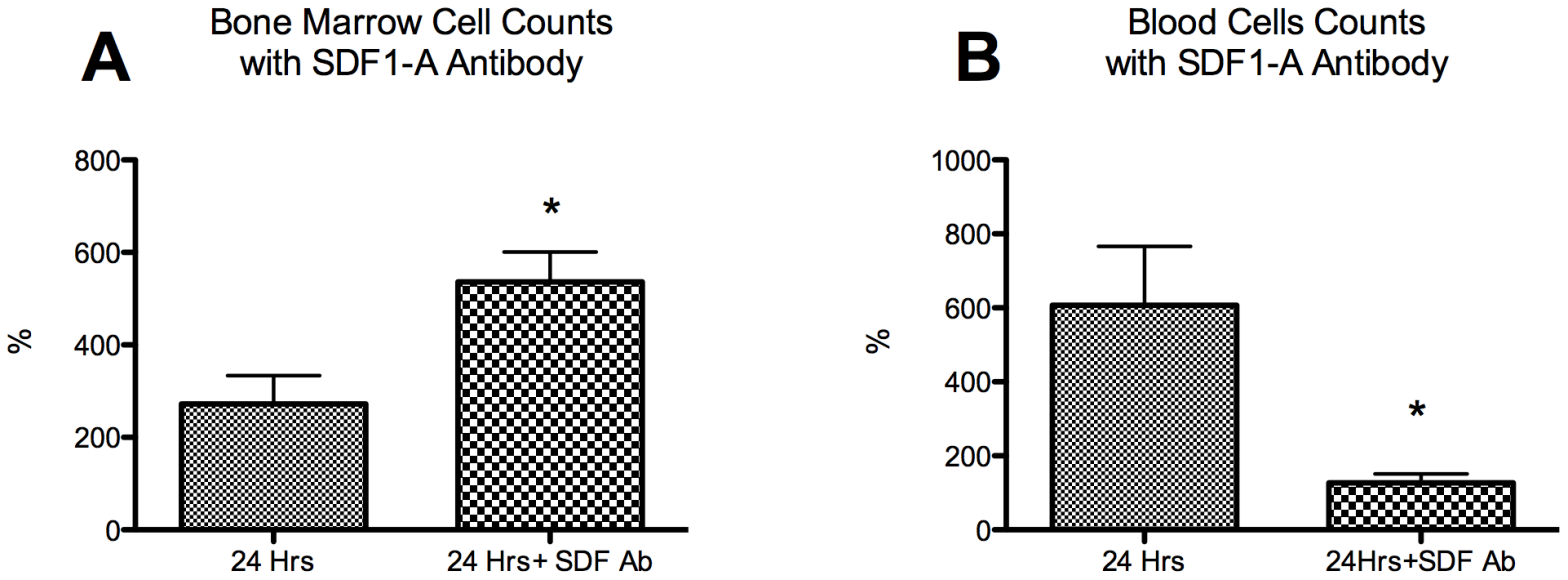
Enumeration of bone marrow and blood Lin−/Sca1+ cell numbers following stroke and SDF1-A Antibody. (A) Twenty-four hour post stroke+SDF1-A antibody mice show a marked increase in the number of Lin−/Sca1+ cells present in the bone marrow compared to controls. (B) Twenty-four hour post stroke mice showed a marked decrease in the mobilization of these cells to the blood. * P<0.05.

Lin−/Sca1+ cells failed to mobilize to the peripheral blood following stroke+SDF1-A antibody (Figure 3), despite continued up regulation in the bone marrow (bone marrow cell counts with stroke+SDF1-A antibody: 536±65% versus stroke+placebo: 272±60%, p<0.05; blood cell counts = SDF1-A antibody: 127±24% versus stroke+placebo: 607±159%, p<0.05).

### Does SDF1-A Blockade Abrogate Homing?

**Figure 4:**
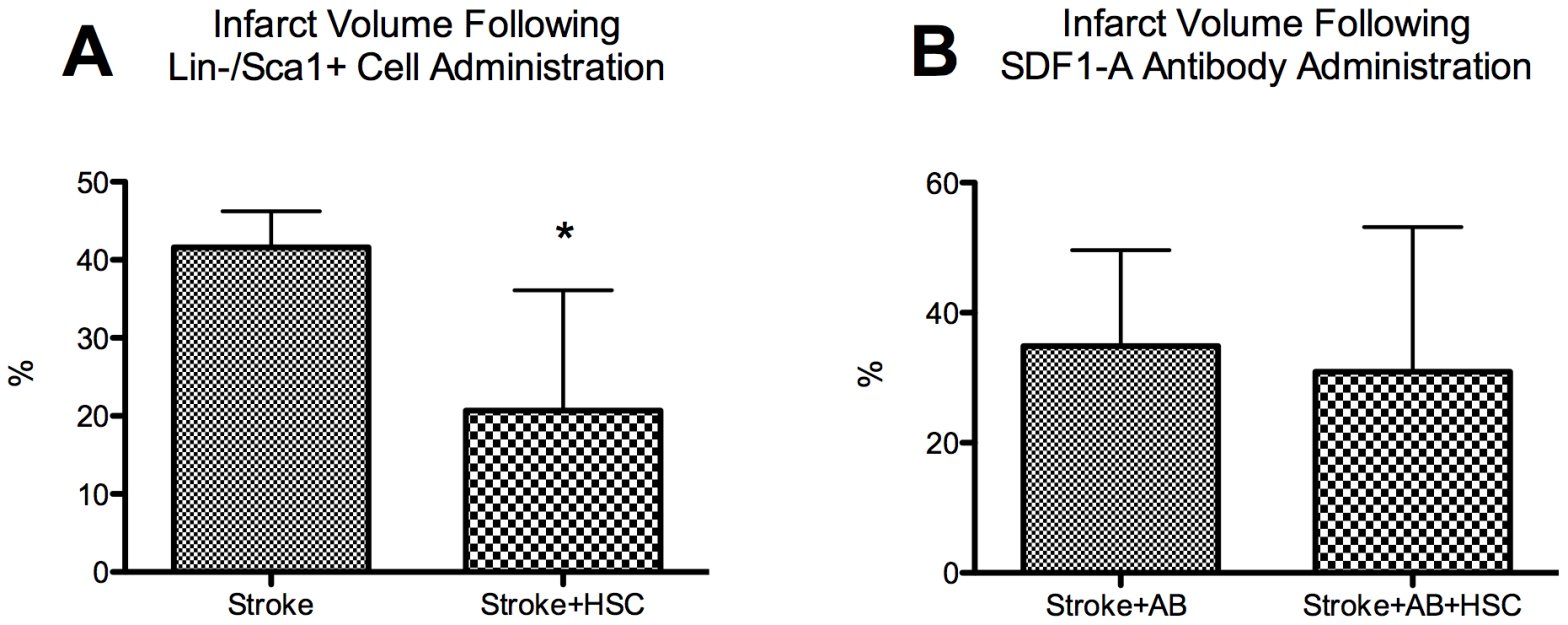
Administration of Lin−/Sca1+ cells post stroke. A. Administration of Lin−/Sca1+ cells post stroke significantly reduced infarct volume at 24 hours post stroke (*P<0.05). B. Administration of the Lin−/Sca1+ cells with an SDF1-A antibody (AB) no longer demonstrates a beneficial reduction in the infarct volume (p = NS).

**Figure 5:**
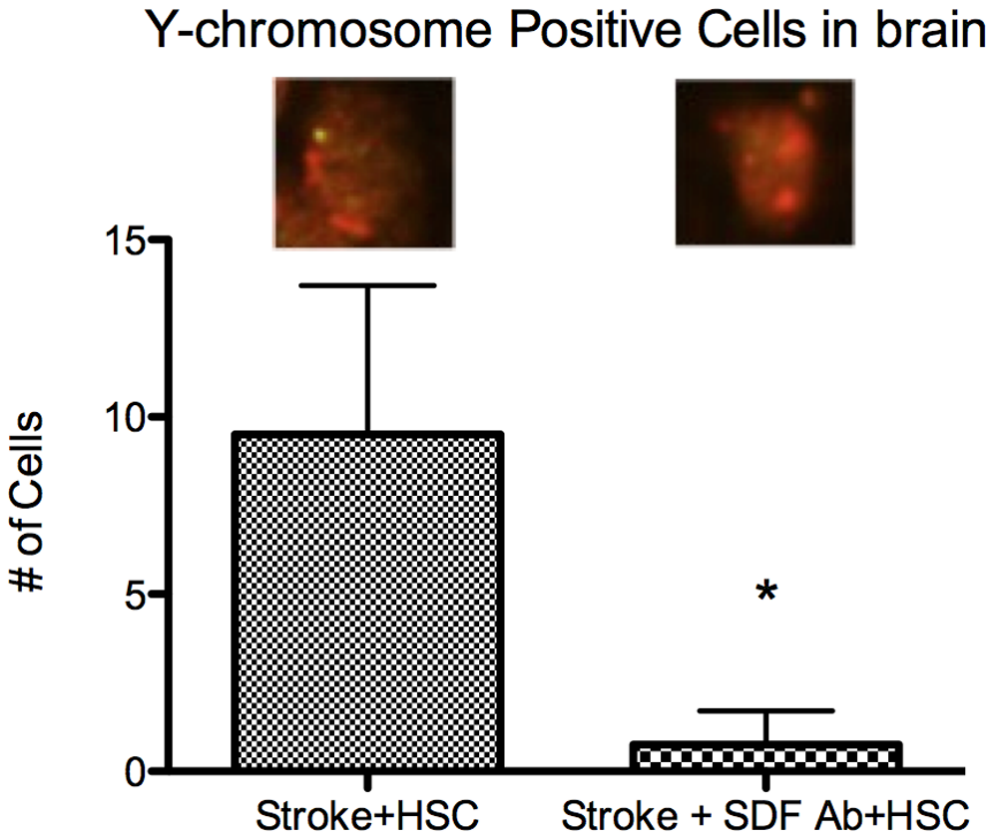
Y chromosome Positive cells in brain. Male Lin−/Sca1+ cells injected IV post stroke were counted in female ischemic hemisphere at 24 hours post stroke; Animals which received SDF1-A antibody in addition to the IV Lin−/Sca1+ cells, had a significantly lower number of cells detected in the brain by FISH (*P<0.05). Green = Y chromosome.

Administration of exogenous Lin−/Sca1+ cells resulted in a significant reduction in infarct volume at 24 hours (Figure 4A) (42±5% versus 21±15%, stroke+placebo versus stroke+ Lin−/Sca1+ cells, p<0.05). In contrast, administration of the SDF1-A antibody concomitant to the injection of exogenous Lin−/Sca1+ cells (Figure 4B) prevented any reduction in infarct volume (35±15 versus 31±22%; stroke+SDF1-A antibody+placebo vs stroke+SDF1-A antibody+ Lin−/Sca1+ cells, p = NS). Furthermore, FISH analysis demonstrated that administration of male Lin−/Sca1+ cells to female mice upon reperfusion resulted in identification of Y chromosome positive cells in the ischemic hemisphere (Figure 5). However, this effect was abrogated when the male Lin−/Sca1+ cells were administered concomitant to an SDF1-A antibody (10±4 versus 0.7±1, P<0.05).

## Discussion

Recent studies have demonstrated the ability of HSC/HPC to home to an area of injury (in ischemic hind limb, [40] cardiac disease [41] and liver [42]). While, the mechanism involved HSC/HPC recruitment to the area of injury is poorly defined, SDF1-A has been implicated in the homing process. [22], 36,37 The results of the studies presented herein suggest that recruitment of Lin−/Sca1+ cells to stroked brain occurs along an SDF1-A pathway.

Lin−/Sca1+ cell counts indicate that bone marrow Lin−/Sca1+ cell production increased post stroke, followed by Lin−/Sca1+ cell mobilization to the peripheral blood (Figure 1). Several studies have shown that Lin−/Sca1+ cells mobilize from the bone marrow to the peripheral blood in response to injury [27] and that these cells contribute to recovery. [43] However, the mechanism involved in mobilization and consequent homing following stroke has yet to be investigated. We chose to perform evaluations at 4 hours and at 24 hours. These time points were specifically chosen as 24 hours represents a standard time point across the majority of murine intraluminal filament studies. Four hours was chosen as it reasonably reflects the time window for current Class I evidence based clinical stroke intervention with IV tPA (with centers using either a 3 or 4.5 hour window). A more expansive number of time point evaluations would be of interest and our study is limited by containing only these two time points, however, logistic and economic limitations prevented a more detailed time point analysis.

Once confirmation of Lin−/Sca1+ cell up-regulation and mobilization was obtained we then sought to determine whether Lin−/Sca1+ cells navigate to the area of cerebral ischemia in response to an SDF1-A gradient. Serum SDF1-A levels did not achieve significance until 24 hours post stroke surgery (Figure 2A). This correlated well with a significant increase in production in the bone marrow and mobilization of these cells to the blood at 24 hours. Interestingly, the ischemic hemisphere experienced highly significant elevations in SDF1-A levels within 4 hours (Figure 3), providing a temporal indicator that the source of subsequent serum elevations may be the ischemic brain itself. It may also be that it is this brain to serum to bone marrow gradient that then results in Lin−/Sca1+ cell homing to the ischemic hemisphere.

Neutralizing SDF1-A, through administration of an SDF1-A antibody, prevented mobilization of Lin−/Sca1+ cells from bone marrow to blood (Figure 3). Leading to the effective sequestration of Lin−/Sca1+ cells within the bone marrow following stroke and a significantly reduced number of Lin−/Sca1+ cells in the blood. However, antibody administration did not prevent continued bone marrow upregulation. This suggests that initial activation of Lin−/Sca1+ cell production likely occurs through an alternate signaling pathway, but that subsequent movement of Lin−/Sca1+ cells from bone marrow to blood, and then from blood to ischemic hemisphere appears to be dependent upon an SDF1-A. To ensure these findings are not an unintended epiphenomenon resulting from alternative effects of the antibody, alternative methods of SDF1-A abrogation should be evaluated before this phenomenon can be conclusively tied to an SDF1-A pathway alone. However, this initial implication is encouraging that an SDF1-A dependent pathway is critical to Lin−/Sca1+ cell homing following stroke. Shichinohe et al. provided additional support for the importance of the SDF1-A pathway when they evaluated parenchymally injected BSMC migration following rodent stroke. [37] They observed significant mitigation of the migratory response in CXCR4, a major receptor for SDF1-A, knockout mice. [37] Wang et al. further supported an SDF1-A critical pathway when they demonstrated that GFP labeled exogenous (IV administered) BSMCs homed to ischemic brain in rats and that this homing was abrogated with the administration of a CXCR4 antibody. [36] However, (1) both of these studies evaluated CXCR4 based blocking mechanisms, making the assessment SDF1-A specific blocking of additional value; (2) both of these studies relied solely on exogenous BSMCs, which may behave different than endogenous cells; (3) neither of these studies evaluated the bone marrow and blood response to cerebral ischemia, which is a crucial component to understanding the overall pathway of hematogenous-based stroke recovery mechanisms; and (4) both of these studies used rat models of stroke. This last point is particularly of interest, as Steiner et al. demonstrated no homing of exogenously (IV) administered human MSCs in a murine model of stroke, despite confirmation of cell migration to peripheral organs. The relevance of these mechanisms to murine stroke is crucial as most preclinical restorative therapy work has previously, and currently continues, in mice.

The data contained in the current study suggest that brain tissue of stroked mice does generate SDF1-A and thereby recruits Lin−/Sca1+ cells along an SDF1-A gradient to the area of ischemic brain. That cerebral infarct volume reduction, identified following exogenous Lin−/Sca1+ cell administration, was abrogated when Lin−/Sca1+ cell administration occurred concomitant to SDF1-A antibody administration provides further support to this hypothesis (Figure 4). While this effect may be secondary to unintended and unaccounted for effects of the SDF1-A antibody, it appears likely that the reduction in benefit was at least in part due to the prevention of appropriate homing of the Lin−/Sca1+ cells along the SDF1-A gradient. Previous investigators have demonstrated improvement in stroke volumes with exogenous BSMC administration, [36] however, the data contained herein provides crucial evidence that homing occurs in a murine model, that it provides a reduction in infarct volume, and is dependent on the SDF1-A pathway.

Further support for an SDF1-A dependent pathway was evidenced when SDF1-A antibody administration prevented detection of significant numbers of Y chromosome positive Lin−/Sca1+ cells in the brain, despite evidence of Y chromosome Lin−/Sca1+ cell homing without the SDF1-A antibody (Figure 5). These data lends further credence to the hypothesis that the homing mechanism occurs through an SDF1-A dependent pathway. This finding also implies that SDF1-A’s effect is not limited to its potential role in mobilization from the bone marrow to the blood, but also through a direct effect in guiding circulating Lin−/Sca1+ cells homing to the ischemic brain, as the male Lin−/Sca1+ cells were exogenously administered, and so should not be subject to sequestration in the bone marrow. Male mice were used for the initial portion of this study to avoid contribution of the estrogen/estrogen receptor axis towards SDF1-A production. However, female mice were utilized for this homing analysis in order to facilitate tracking the Y chromosome positive cells in the brain. It should be noted that gender differences exist with respect to SDF1-A production, especially in cardiac reperfusion (I/R) studies. Huang et al [44] showed that despite similar baseline SDF1-A levels in male and female rats, the female rats had higher levels of SDF1-A production following I/R. Subsequent interpretation should be conscientious of this axis, however, it is our intent to merely utilize the chromosome difference between genders as a tool to provide some additional insight into SDF1-A mediation of homing. Additionally, such cross gender analysis has been well established in the stroke literature. [44], [45].

In ischemic cardiovascular disease, SDF1-A therapy has led to increased cell survival, neovascularization and tissue repair; [46] Blocking the SDF1-A/CXCR4 axis with AMD3100 (a CXCR4 antagonist) has been shown to cause a decrease in the number of cells homing to an area of injury and an exacerbated apoptotic cell death. [47] In our study, SDF1-A was blocked using an anti-SDF1-A antibody rather than using an antibody directed to its receptor, CXCR4, as previously performed by Wang et al. [36] SDF1-A has recently been shown to bind to another receptor, CXCR7. [20] Blocking of the CXCR4 receptor alone provides a potential alternative mechanism of effect for SDF1-A. Given Wang et al. and Shichinohe et al.’s prior work it appears likely that CXCR4 is crucial to the overall pathway, however, as they did not evaluate blood and bone marrow for possible sequestration, it is possible that the peripheral effects we observed are due to either CXCR4 or CXCR7. Further evaluation of the separate SDF1-A receptors will better elucidate which of those receptors play a critical role in the various components of post-stroke homing.

Our results suggest that local incremental increases in SDF1-A facilitate homing of Lin−/Sca1+ cells, and that neutralizing SDF1-A with an antibody abolished the homing of Lin−/Sca1+ cells to stroked brain. Cerebral ischemia leads to an increase in SDF1-A in the ischemic hemisphere, as well as increased production of Lin−/Sca1+ cells in the bone marrow. These cells then mobilize to the blood, apparently in response to an SDF1-A gradient. Furthermore, SDF1-A appears to play a critical role in modulating Lin−/Sca1+ cell migration to the site of the stroke itself and that administration of an SDF1-A neutralizing antibody impaired both the mobilization of Lin−/Sca1+ cells to the blood, as well as the homing of exogenous Lin−/Sca1+ cells to ischemic brain. These data suggest a critical role for SDF1-A following cerebral ischemia; however further studies are required to more clearly delineate the mechanisms by which SDF1-A exerts its effects.
